# Role of GARP family transcription factors in the regulatory network for nitrogen and phosphorus acquisition

**DOI:** 10.1007/s10265-023-01513-0

**Published:** 2024-01-08

**Authors:** Naohiko Ohama, Shuichi Yanagisawa

**Affiliations:** Plant Functional Biotechnology, Agro-Biotechnology Research Center, Graduate School of Agricultural and Life Sciences, The University of Tokyo, Yayoi 1-1-1, Bunkyo-ku, Tokyo, 113-8657 Japan

**Keywords:** Dimerization, GARP family protein, Nitrogen, Nutrient acquisition, Phosphorus

## Abstract

The GARP (Golden2, ARR-B, Psr1) family proteins with a conserved DNA-binding domain, called the B-motif, are plant-specific transcription factors involved in the regulation of various physiological processes. The GARP family proteins are divided into members that function as monomeric transcription factors, and members that function as transcription factors in the dimeric form, owing to the presence of a coiled-coil dimerization domain. Recent studies revealed that the dimer-forming GARP family members, which are further divided into the PHR1 and NIGT1 subfamilies, play critical roles in the regulation of phosphorus (P) and nitrogen (N) acquisition. In this review, we present a general overview of the GARP family proteins and discuss how several members of the PHR1 and NIGT1 subfamilies are involved in the coordinated acquisition of P and N in response to changes in environmental nutrient conditions, while mainly focusing on the recent findings that enhance our knowledge of the roles of PHR1 and NIGT1 in phosphate starvation signaling and nitrate signaling.

## Introduction

Being sessile organisms, plants need to cope with various environmental challenges at the site of germination. To overcome adverse environmental conditions, plants have developed complicated gene regulatory networks that optimize their growth in response to the external environment (Li et al. 2021a; Waadt et al. 2022). Transcription factors (TFs) are the main regulators of gene regulatory networks. The *Arabidopsis thaliana* genome encodes 2,296 TFs, which are classified into 58 families (Jin et al. 2017). Because of their functional importance, TFs have been considered as one of the main targets of plant research. According to previous studies, functional differences occur not only among the different TF families but also among members of the same TF family. The GARP (Golden2, ARR-B, Psr1) family of TFs is such an example. The GARP family was first identified by Riechmann et al. (2000), when Arabidopsis TFs were systemically identified using whole-genome information. The family name GARP is derived from the names of its constituent members identified at the early stage of GARP research, namely, the GOLDEN 2 (G2) protein of maize (Hall et al. 1998), Arabidopsis RESPONSE REGULATOR-B (ARR-B) protein (Imamura et al. 1999), and the PHOSPHATE STARVATION RESPONSE 1 (PSR1) protein of *Chlamydomonas* (Wykoff et al. 1999); these proteins are involved in chloroplast development, cytokinin signaling, and phosphate starvation response, respectively. Subsequent studies revealed a variety of functions of GARP family members, including plant hormone signaling, circadian clock regulation, organ development, and nutrient acquisition (Safi et al. 2017), highlighting that these proteins play vital physiological roles throughout the plant life cycle. Intriguingly, recent studies revealed that the interplay of two large subfamilies of the GARP family, PHR1 and NIGT1 subfamilies, generates a sophisticated regulation of nitrogen (N) and phosphorus (P) acquisition to facilitate plant adaptation to the fluctuating nutrient conditions. The current review presents a general overview of the GARP family proteins and discusses how the PHR1 and NIGT1 subfamily proteins cleverly regulate N and P acquisition via their interplay. Since most of the previous studies on GARP family proteins were conducted in Arabidopsis, the genes and proteins included in this review are mostly of Arabidopsis origin, unless specified otherwise.

## Definition and structural characteristics of GARP family proteins

GARP family proteins have a characteristic DNA-binding domain, called the B-motif (Imamura et al. 1999). Because of sequence similarity between the B-motif and the DNA-binding domain of MYB TFs, the GARP family proteins are often confused with MYB-like proteins. Nuclear magnetic resonance spectroscopy of the ARR10–DNA complex demonstrated that the GARP B-motif and the MYB DNA-binding domain exhibit a similar three-dimensional (3D) structure (Hosoda et al. 2002). However, phylogenetic analysis showed that GARP family proteins are distinct from MYB-related proteins (Fitter et al. 2002). Consistent with the result of this analysis, some critical amino acids in the MYB DNA-binding domain are not conserved in the B-motif (Safi et al. 2017). Therefore, GARP family proteins are considered to be plant-specific TFs that are distinguishable from the widely conserved eukaryotic MYB proteins (Riechmann et al. 2000).

The structure of all domains, except the DNA-binding domain, of GARP family proteins is highly diverse (Safi et al. 2017). However, GARP family proteins are broadly classified into monomeric members and members that form dimers owing to the presence of a coiled-coil dimerization domain (Safi et al. 2017). Since many TFs possess a dimerization domain for homo- or heterodimerization, which is often required for DNA binding (Amoutzias et al. 2008), the DNA-binding and dimerization domains are generally shared among all members of each TF family. Therefore, the presence of both monomeric members and dimer-forming members is characteristic of the GARP family.

**Fig. 1:**
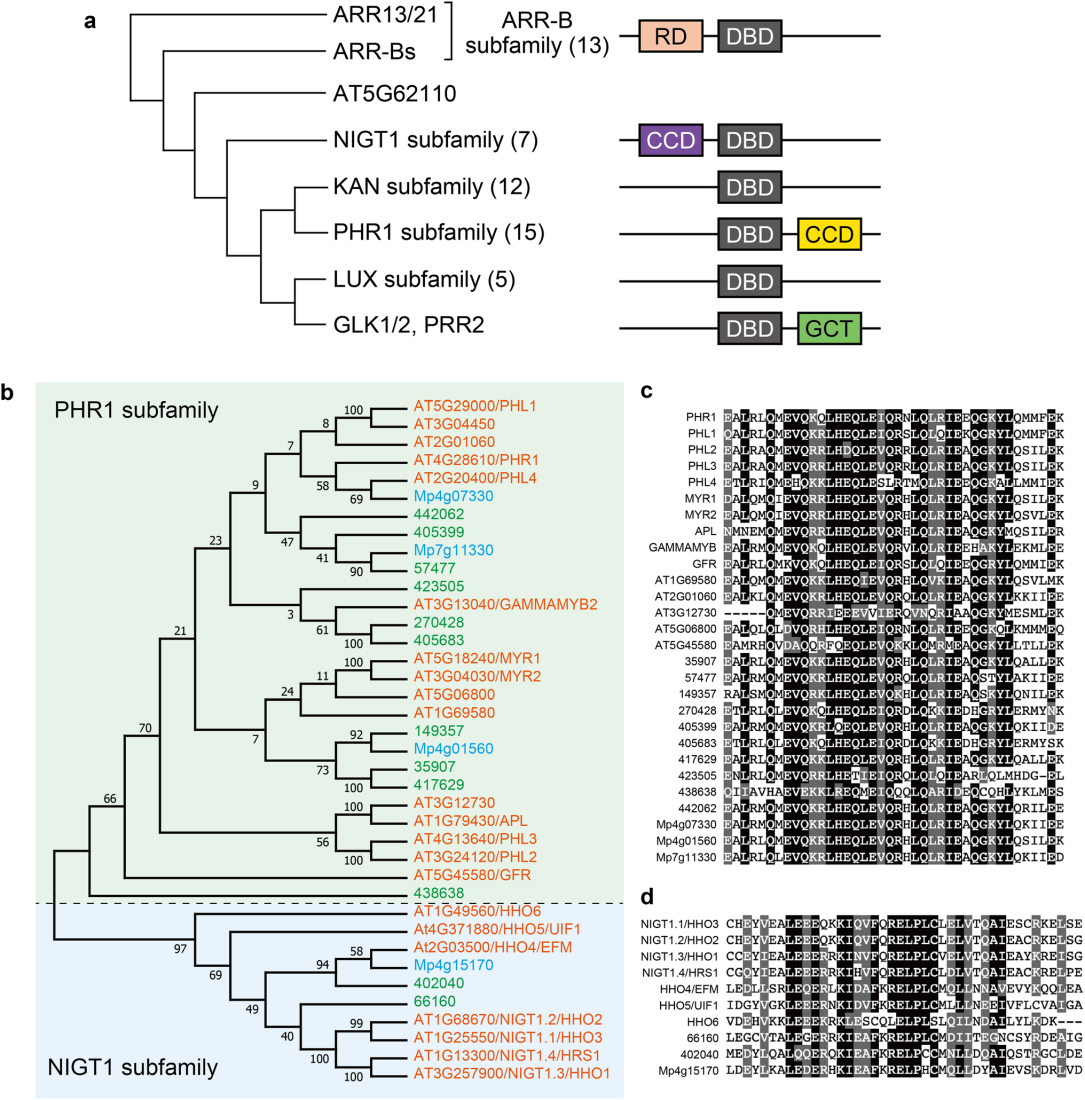
Phylogenetic relationship and structure of GARP family proteins. **a** A phylogenetic tree (left) and representative structure (right) of Arabidopsis GARP family proteins. Phylogenetic analysis was performed with the SALAD Database (Mihara et al. 2010). Numbers shown in parentheses indicate the number of proteins included in each subfamily. Each representative structure indicates a domain that is conserved among the subfamily members. CCD, Coiled-coil domain; DBD, DNA-binding domain; GCT, GLK/C-terminal box; RD, Receiver domain. **b** Phylogenetic tree constructed using the amino acid sequences of the PHR1 and NIGT1 subfamily proteins of *Marchantia polymorpha* (blue), *Selaginella moellendorffii* (green), and *Arabidopsis thaliana* (orange). Proteins in green and blue background belong to the PHR1 and NIGT1 subfamilies, respectively. The phylogenetic tree was generated using MEGA11 (Tamura et al. 2021). The bootstrap values were calculated based on 1,000 replications. **c** and **d** Sequence alignment of the CCD of PHR1 (**c**) and NIGT1 (**d**) subfamily members. Sequences with > 80% identity or similarity are shaded in gray and black

The monomeric GARP family TFs are further classified into four subfamilies, consisting the ARR, GOLDEN 2-LIKE (GLK), LUX ARRHYTHMO (LUX), and KANADI (KAN) subfamilies, which were defined by the presence of several unique amino acid sequence motifs, while GARP family proteins that function as dimeric TFs containing a coiled-coil domain (CCD) are classified into two subfamilies, the PHOSPHATE STARVATION RESPONSE 1 (PHR1) and NITRATE-INDUCIBLE, GARP-TYPE TRANSCRIPTIONAL REPRESSOR 1 (NIGT1) subfamilies (Fig. 1a; Safi et al. 2017). These subfamilies differ phylogenetically and regulate different physiological processes (Safi et al. 2017). Arabidopsis contains 56 GARP family proteins, 55 of which belong to the ARR (13 proteins), GLK (3 proteins), LUX (5 protein), KAN (12 proteins), PHR1 (15 proteins), and NIGT1 (7 proteins) subfamilies, while 1 GARP family protein (AT5G62110) is not assigned to any subfamily, because it lacks the N-terminal half of the DNA-binding domain and does not possess any characteristic amino acid motifs (Fig. 1a). Furthermore, PSEUDO-RESPONSE REGULATOR 2 (PRR2) is a hybrid protein that harbors amino acid motifs conserved among the proteins of ARR-B and GLK subfamilies. Although generally considered as a member of the ARR subfamily, PRR2 is classified into the GLK subfamily in this review, based on the results of phylogenetic analysis.

## Physiological functions of monomeric GARP TFs

The monomeric GARP family TFs mediate several physiological responses in plants, based on the evidence available mainly in Arabidopsis. GARP family proteins belonging to the ARR-B subfamily are the critical regulators of cytokinin signaling (Ferreira and Kieber 2005; Hwang et al. 2012). The cytokinin signal is transferred from the membrane-localized cytokinin receptors (AHKs) to histidine phosphotransfer proteins (AHPs) via a phosphorylation relay. Then, the activated AHPs phosphorylate ARR-B proteins at their N-terminal receiver domain (Argyros et al. 2008; Yokoyama et al. 2007). Consistent with the fact that ARR-B proteins are responsible for the expression of most cytokinin-inducible genes (Argyros et al. 2008; Yokoyama et al. 2007), the Arabidopsis *arr1 arr10 arr12* triple mutant exhibited severe defects in its physiological and transcriptional responses to the exogenously applied cytokinin and showed various abnormalities in cell division and organ differentiation. On the other hand, GLKs have been shown to play an essential role in chloroplast development in several plant species, including Arabidopsis, tomato (*Solanum lycopersicum*), and rice (*Oryza sativa*) (Nguyen et al. 2014; Wang et al. 2013; Waters et al. 2009). GLKs are required for the expression of nuclear-encoded photosynthetic proteins related to the light-harvesting complex and chlorophyl biosynthesis (Fitter et al. 2002; Waters et al. 2009). Thus, the functionally deficient mutants of *GLKs* show pale-green leaves with low chlorophyll content and a small number of chloroplasts with developmentally abnormal thylakoid membrane structure (Fitter et al. 2002). Arabidopsis and tomato possess two functionally redundant GLK paralogs (GLK1 and GLK2), although the fruit tissue predominantly expresses *GLK2* (Fitter et al. 2002; Nguyen et al. 2014; Powell et al. 2012). Therefore, Arabidopsis and tomato *glk2* mutants, unlike the Arabidopsis *glk1* mutant and tomato *GLK1* co-suppression lines, show reduced chloroplast development specifically in fruits (Fitter et al. 2002; Nguyen et al. 2014; Powell et al. 2012). LUX (of the LUX subfamily) functions as a DNA-binding component in the Evening Complex (EC), which is a transcriptional repressor complex and a core regulator of the plant circadian clock (Hazen et al. 2005; Silva et al. 2020). As a component of the EC, LUX represses the expression of clock-regulated genes and therefore is responsible for maintaining circadian oscillation. *LUX* expression is induced during nighttime and is under the control of a negative autoregulatory feedback loop, because LUX directly binds to its own promoter (Helfer et al. 2011). A phylogenetically close homolog of LUX, named BROTHER OF LUX ARRHYTHMO (BOA), is also involved in the regulation of circadian oscillation (Dai et al. 2011). KAN subfamily members are involved in organ patterning through the establishment of abaxial/adaxial polarity. In Arabidopsis, *KAN1–4* genes are mainly expressed in the abaxial region of tissues and are responsible for the maintenance of abaxial identity (Eshed et al. 2004; Kerstetter et al. 2001; McAbee et al. 2006). The spatial expression pattern of these *KAN* genes regulates auxin signaling to induce KAN-mediated organ patterning (Izhaki and Bowman 2007; Merelo et al. 2013). In addition, *KAN1–4* genes are required for the normal development of vascular tissues and reproductive organs (Emery et al. 2003; Eshed et al. 2001; Ilegems et al. 2010; Kerstetter et al. 2001). Since not all members of the monomeric GARP TF subfamilies have been characterized yet, further studies are needed to reveal the functions of these proteins. However, at this stage, members belonging to the same subfamily of monomeric GARP TFs appear to play closely related roles in the same physiological process.

## Dimerization domains and DNA recognition of dimeric GARP TFs

Many members of the two subfamilies of dimeric GARP TFs, PHR1 and NIGT1 subfamilies (Fig. 1b), have been shown to play critical roles in regulating nutrient responses (discussed below); however, some members of the PHR1 and NIGT1 subfamilies are suggested to play vital roles in the developmental processes. ALTERED PHLOEM DEVELOPMENT (APL), a member of the PHR1 subfamily, is a critical regulator for the definition of phloem identity (Abe et al. 2015; Bonke et al. 2003; Kim et al. 2021; Kondo et al. 2016; Zhao et al. 2011), but the role of APL in nutrient responses has yet to be shown. HYPERSENSITIVITY TO LOW PI-ELICITED PRIMARY ROOT SHORTENING 1 HOMOLOGUE 4 (HHO4)/EARLY FLOWERING MYB PROTEIN (EFM) and HHO5/ULTRAPETALA 1 INTERACTING FACTOR 1 (UIF1) are NIGT1 subfamily members involved in the flowering and floral organ development, respectively (Moreau et al. 2016; Yan et al. 2014).

PHR1 subfamily proteins possess a coiled-coil-type dimerization domain downstream of their DNA-binding domain (Fig. 1a, c). Because of this structural feature, Lundmark et al. (2011) referred to the PHR1 subfamily as the GARP coiled-coil family. By contrast, the NIGT1 subfamily proteins possess another coiled-coil domain for dimerization upstream of their DNA-binding domain (Fig. 1a, d) (Safi et al. 2017; Ueda et al. 2020b). The coiled-coil dimerization domain of NIGT1 proteins, which was referred to as the hydrophobic and globular domain by Li et al. (2021b), shares no amino acid sequence similarity with that of PHR1 subfamily proteins and therefore is easily distinguishable.

Phylogenetic analysis showed that PHR1-like and NIGT1-like proteins show a similar domain structure in liverwort (*Marchantia polymorpha*) and spike moss (*Selaginella moellendorffii*) (Fig. 1b–d). Furthermore, the domain structure, dimerization domain, and physiological role of *Chlamydomonas* PSR1 are similar to those of PHR1 (Rubio et al. 2001; Wykoff et al. 1999). Therefore, the physiological functions of PHR1 and NIGT1 subfamily proteins might be evolutionally conserved. Although many studies revealed that both PHR1 and NIGT1 subfamily proteins are closely associated with nutrient responses, the PHR1 and NIGT1 subfamilies do not constitute a monophyletic group. Hence, it was hypothesized that the ancestors of PHR1 and NIGT1 subfamilies evolved their respective dimerization domains independently (Safi et al. 2017). Consistently, Ueda et al. (2020b) showed that the NIGT1 subfamily proteins dimerize among themselves (to form homo- and heterodimers) but do not dimerize with PHR1 subfamily proteins.

**Fig. 2:**
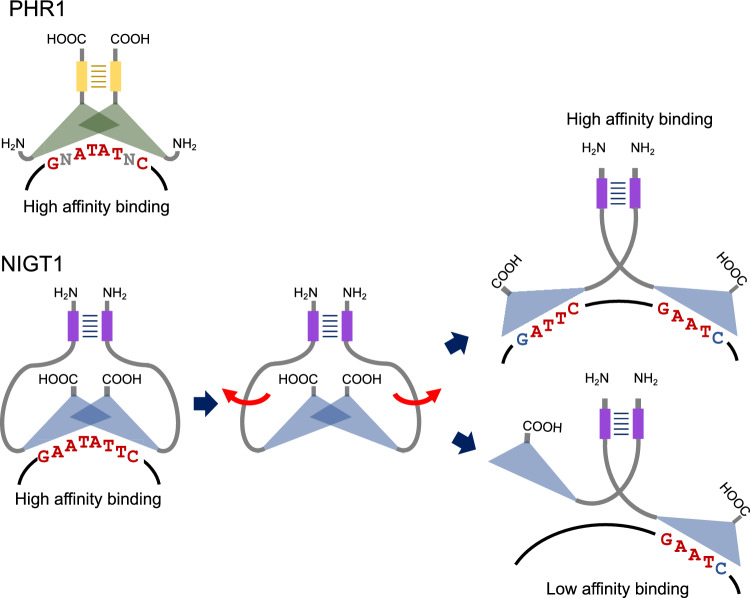
A model depicting the DNA-binding modes of PHR1 and NIGT1 proteins. PHR1 and NIGT1 form dimers using the coiled-coil-type dimerization domains (yellow and purple rectangles, respectively) and bind to palindromic DNA sequences (-GNATATNC- and -GAATATTC-, respectively). In this DNA-binding mode, two DNA-binding domains (green and blue triangles) align closely and symmetrically. The NIGT1 dimer also binds to the NIGT1-binding sequence when present singly (-GAATC-) or as a pair (e.g., -GATTC-N_38_-GAATC-). A conformational change in the linker region that connects the dimerization domain with the DNA-binding domain allows NIGT1 to flexibly position the two DNA-binding domains. In this DNA-binding mode, two DNA-binding domains independently recognize the target sequences, including the additional cytosine (shown in blue), compared with the palindromic target sequence

The dimerization domains of both PHR1 and NIGT1 subfamily proteins are essential for DNA recognition. Deletion of the dimerization domain of PHR1 abolished its DNA-binding ability (Rubio et al. 2001). Similarly, high-affinity DNA binding of NIGT1 proteins requires dimerization (Ueda et al. 2020b). However, mutations in the dimerization domain of NIGT1 did not completely abolish its DNA-binding activity, indicating that the NIGT1 DNA-binding domain of monomeric NIGT1 is still able to recognize its target sequence. Based on these observations, it was hypothesized that PHR1 and NIGT1 proteins use different mechanisms to bind to DNA (Fig. 2) (Yanagisawa 2013).

The PHR1-binding sequence (P1BS) is the -GNATATNC- palindromic sequence, in which N indicates any nucleotide. Thus, the dimerization of PHR1 proteins likely results in the formation of a rigid 3D structure with two closely and symmetrically aligned PHR1 DNA-binding domains, which are necessary for DNA-binding activity. Previously, comparison between the DNA-binding domains of PHR1 and ARR10 led to the speculation that well-conserved amino acid residues in the two DNA-binding domains are involved in the nucleotide recognition of one half of the P1BS (-GNAT-) (Yanagisawa 2013). Although the leucine residue involved in the recognition of cytosine in the ARR10-binding sequence (-AATCT-) is also conserved in PHR1, PHR1 does not seem to use this residue to bind to DNA, probably because the access of this residue is structurally inhibited by the close positioning of two DNA-binding domains. Thus, each protein component of the GARP monomers and dimers shares a similar DNA recognition pattern, although dimerization alters this recognition pattern.

On the other hand, NIGT1 employs two DNA recognition modes to bind to a variety of target sequences (Ueda et al. 2020b) (Fig. 2). One of these modes is similar to that of PHR1, where an NIGT1 dimer binds to a palindromic sequence (-GAATATTC-). Because a mutation in the dimerization domain disrupts the binding of NIGT1 proteins to this palindromic sequence, close positioning of two DNA-binding domains is necessary for this DNA recognition mode. The other DNA recognition mode is unique to NIGT1 proteins, in which the NIGT1 dimer recognizes a single copy and two separated copies of the NIGT1-binding sequence (-GAATC-) with low and high affinities, respectively. Although structural details should be verified in a future study, the two DNA-binding domains of an NIGT1 dimer independently recognize the target sequence much like a monomeric GARP protein. The NIGT1-binding sequence in this type of DNA recognition (-GAATC-) shares an AATC motif with the ARR10-binding sequence (-AATCT-), suggesting that the 3D structure of the NIGT1–DNA complex in this DNA recognition mode is similar to that of the ARR10–DNA complex. The fact that amino acid residues recognizing these nucleotides in ARR10 are highly conserved in the NIGT1 DNA-binding domain is consistent with this hypothesis (Yanagisawa 2013). Recognition of an additional cytosine in this type of DNA recognition sequence also implies more space between the two NIGT1 DNA-binding domains compared with the binding mode for the -GAATATTC- sequence. Thus, NIGT1 can recognize two distant target -GAATC- sequences with face-to-face (e.g., -GATTC-N_38_-GAATC- in the *NRT2.1* promoter) (Ueda et al. 2020b). This difference implies that the structure of the linker region between the dimerization and DNA-binding domains is flexible for the appropriate positioning of the DNA-binding domain, depending on the target sequences. Further structural analyses of the NIGT1 dimer may reveal molecular details of how NIGT1 utilizes the two distinct DNA-binding modes.

The dimerization of TFs allows them to recognize longer and more complex sequences, which enables plants to develop complicated transcriptional regulatory networks. The formation of heterodimers can alter the DNA-binding affinity and sequence specificity of each constituent monomer, depending on their characteristics. Therefore, in some cases, the functions of dimerized TFs can be modified by regulating the expression ratios and dimerization efficiency of the participating monomers. In the case of MYB-RELATED PROTEIN 1 (MYR1) and MYR2, members of the PHR1 subfamily, alternative splicing was shown to affect their dimerization efficiency by altering the sequence of the dimerization domain (Zhao and Beers 2013), thus perhaps fine-tuning downstream gene expression. Both PHR1 and NIGT1 subfamily proteins form heterodimers with other family members exhibiting different expression patterns and functions (Ueda et al. 2020b; Wang et al. 2023). Future studies focusing on heterodimer formation may reveal new regulatory roles of dimeric GARP TFs.

## Physiological roles of PHR1 subfamily members in P acquisition

In the past two decades, many studies have revealed that plants precisely control N and P acquisition via a transcriptional regulatory network composed of multiple TFs (Helliwell 2023; Li et al. 2021b; Sega and Pacak 2019; Ueda et al. 2021). PHR1 and NIGT1 subfamily members play essential roles in this network. PHR1 and its homologs have been well characterized as essential positive regulators of phosphate uptake and phosphate starvation responses in Arabidopsis, rice, and several other plant species (Sega and Pacak 2019). Since plants acquire P from the soil as phosphate, the phosphate uptake and phosphate starvation responses are coordinately regulated by PHR1 and its homologs.

PSR1, which was the first PHR1 subfamily member to be reported, was identified in a screening of *Chlamydomonas* mutants showing defective acclimatization to phosphate limitation (Shimogawara et al. 1999; Wykoff et al. 1999). The *psr1* mutant showed no increase in the rate of phosphate uptake upon phosphate depletion, resulting in a rapid decline in photosynthetic activity and growth after transfer to phosphate-limited conditions. Subsequently, Arabidopsis PHR1 was identified through mutant screening with a GUS reporter line, in which *GUS* expression was driven by a phosphate starvation-inducible gene promoter (Rubio et al. 2001). PHR1 functions redundantly with PHL1, which is most closely related to PHR1 in Arabidopsis (Fig. 1b), controlling a large part of the transcriptional response to phosphate starvation. Consequently, the *phr1 phl1* double mutant does not exhibit the typical physiological responses to phosphate starvation, such as anthocyanin accumulation, root hair elongation, and increased root/shoot ratio, and shows severe growth defects under phosphate-deficient conditions (Bustos et al. 2010). PHR1 homologs, PHL2 and PHL3, are also involved in the phosphate starvation response (Sun et al. 2016). PHR1 and PHLs share the dimerization domain and the DNA-binding sequence; however, PHR1/PHL1 and PHL2/PHL3 function as distinct modules to regulate plant development and transcriptional responses, because they do not physically interact with each other (Wang et al. 2023). The distinct roles of PHR1/PHL1 and PHL2/PHL3 in the phosphate starvation response may be related to the distant position of PHR1/PHL1 and PHL2/PHL3 in the phylogenetic tree (Fig. 1b), implying that many members of the PHR1 subfamily may be involved in phosphate-related physiological processes.

Interestingly, PHR1 was found to not only directly activate the phosphate starvation-related genes but also suppress the immune response-related genes. This PHR1-mediated regulation balances resource allocation between phosphate acquisition and immunity and modulates root microbial community. Under phosphate starvation conditions, plants allow colonization by beneficial microorganisms, such as mycorrhizal fungi, to promote phosphate uptake. A recent study showed that PHR1 activates the genes encoding the immune response-suppressing rapid alkalinization factor peptides to balance phosphate acquisition and immune response in phosphate-deficient environments (Tang et al. 2022). Therefore, PHR1 plays a critical role in controlling the overall physiological responses of plants to enable their survival in phosphate-limited environments.

Unlike PSR1 activity, which is regulated at the transcriptional level (Wykoff et al. 1999), PHR1 activity is post-translationally regulated in response to the phosphate nutrient status. SYG1/PHO81/XPR1 (SPX) proteins interact with PHR1 in an inositol phosphate (InsP)-dependent manner to form the PHR1–InsP–SPX ternary complex and reduce PHR1 activity (Puga et al. 2014; Wang et al. 2014; Wild et al. 2016). Because the InsP level is correlated with the cellular phosphate level, plants monitor the cellular phosphate status based on InsP concentration (Wang et al. 2021). Consequently, SPX interacts with PHR1 to inhibit the binding of PHR1 to the P1BS-containing promoters under phosphate-sufficient conditions; however, reduction in the cellular phosphate level leads to the dissociation of the PHR1–InsP–SPX ternary complex, which increases PHR1 activity and upregulates genes responsive to phosphate starvation. A recent study showed that NLA, an SPX domain-containing E3 ubiquitin ligase, also interacts with PHR1 in a manner similar to that employed by SPX proteins to regulate PHR1 protein stability depending on the phosphate level (Park et al. 2023). Furthermore, PHR1 activity is also considered to be regulated by SUMOylation (Miura et al. 2005). Arabidopsis PHR1 is SUMOylated by SIZ1 SUMO E3 ligase; however, because of the pleiotropic phenotype of *siz1*, the exact effect of SUMOylation on PHR1 activity remains unclear.

## Physiological roles of NIGT1 subfamily members in N acquisition

The first suggested physiological role of NIGT1 subfamily members was phosphate starvation response, because overexpression of an Arabidopsis *NIGT1* gene, termed as *HYPERSENSITIVITY TO LOW PI-ELICITED PRIMARY ROOT SHORTENING 1* (*HRS1/NIGT1.4*), resulted in altered phosphate starvation response compared with the wild type (Liu et al. 2009). However, NIGT1 subfamily members, including HRS1 and HHO proteins, are mainly associated with N responses (Maeda et al. 2018) and the N starvation response (Kiba et al. 2018; Ueda et al. 2020a). Consistently, the rice *NIGT1* (*OsNIGT1*) gene was identified as the most strongly induced TF gene upon nitrate treatment (Sawaki et al. 2013) and all Arabidopsis *NIGT1* genes are typical nitrate-inducible genes (Maeda et al. 2018). By contrast, Arabidopsis *HRS1* and *HHO* genes are slightly induced by phosphate starvation, because PHR1 and PHL1 weakly activate some (but not all) *NIGT1* promoters (Maeda et al. 2018). Following previous publications (Kiba et al. 2018; Li et al. 2021b; Liu et al. 2023; Maeda et al. 2018; Ueda et al. 2020a), the NIGT1 subfamily members encoded by nitrate-inducible genes are referred to as NIGT1 proteins in this review, whereas those encoded by nitrate-non-inducible genes are referred to as HHO proteins. Both NIGT1 and HHO proteins function as transcriptional repressors, probably owing to the presence of the EAR motif, an interaction domain for co-repressors (Kagale and Rozwadowski 2011).

Recent studies revealed the roles of NIGT1 proteins in nitrate signaling and responses. Although plants acquire nitrate and ammonium as N sources from the soil, soil nitrate is the major N source for most land plants in oxidative environments. Therefore, nitrate is a key N nutrient, and the supply of nitrate to N-starved plants induces rapid reprogramming of the transcriptome, triggering nitrate responses including the activation of genes related to nitrate uptake, N assimilation, and transcriptional regulation. In nitrate signaling, nitrate itself acts as the primary signal that directly binds to and activates NIN-LIKE PROTEIN (NLP) TFs, which perform dual functions by acting as the nitrate sensor as well as a master transcriptional activator at the initial stage of the nitrate response (Konishi and Yanagisawa 2013, 2014; Krapp et al. 2014; Liu et al. 2022; Marchive et al. 2013). On the other hand, NIGT1 proteins, which function directly downstream of the NLP TFs, are responsible for the negative feedback regulation of nitrate signaling. Although the role of NIGT1 proteins in nitrate signaling was initially suggested in rice (Sawaki et al. 2013), detailed analyses were performed using four Arabidopsis homologs (NIGT1.1–1.4), which revealed their roles in the downregulation of nitrate responses (Maeda et al. 2018; Ueda et al. 2020a, b). NIGT1 proteins negatively regulate the expression of many genes activated by nitrate-activated NLP TFs, which are very frequently related to nitrate transport, nitrate assimilation, cytokinin biosynthesis, and abscisic acid degradation (Maeda et al. 2018; Ueda and Yanagisawa 2019). Thus, the NLP and NIGT1 TFs form an incoherent type I feedforward loop to stabilize the expression of the common target genes of NLP and NIGT1 proteins, such as *NITRATE TRANSPOTER2.1* (*NRT2.1*) (Ueda and Yanagisawa 2019, 2023). Furthermore, NIGT1 proteins have been shown to bind to NIGT1 recognition sequences in their gene promoters, indicating that NIGT1 proteins constitute a negative autoregulatory loop to modulate their expression levels during the nitrate response (Maeda et al. 2018; Sawaki et al. 2013). This complicated regulatory system that includes the NLP–NIGT1 transcriptional module likely optimizes nitrate responses under a myriad of different environmental situations. In addition to the downregulation of nitrate responses, the NIGT1 proteins also regulate N starvation responses (Kiba et al. 2018). NIGT1 proteins suppress N starvation-responsive genes when the N nutrient is abundant, whereas *NIGT1* expression is decreased upon nitrate depletion, leading to the de-repression of N starvation-responsive genes, including *NRT2.4* and *NRT2.5*, to enhance nitrate uptake.

According to recent studies, the HHO proteins also play critical roles in N deficiency responses. In the gene regulatory network controlling N deficiency responses in rice, which was identified through a weighted gene co-expression network analysis and GENIE3-based regulatory network analysis, OsHHO3 and OsHHO4 were designated as the strongest candidates for the central regulators of N deficiency responses in rice. Indeed, very recently, OsHHO3 was reported as a transcriptional repressor of three *AMMONIUM TRANSPORTER 1* (*AMT1*) genes responsible for most of the ammonium uptake activity of plants under N-deficient conditions (Liu et al. 2023). AMT1 activity is tightly associated with plant growth in rice, because rice plants grow in paddy fields and prefer ammonium over nitrate as the N source. Like the expression of Arabidopsis *NIGT1*, the expression of *OsHHO3* is repressed under N-deficient conditions. Therefore, rice plants enhance AMT1 activity and ammonium uptake by reducing *OsHHO3* expression upon N deficiency. Interestingly, *OsHHO3* expression level showed a negative correlation with plant biomass and *AMT1* expression in rice cultivars under N-deficient conditions, implying that natural variation in *OsHHO3* expression levels among rice accessions should be utilized to improve the N use efficiency of rice cultivars (Liu et al. 2023).

Recent studies showed that NIGT1 and HHO proteins play key roles in regulating N deficiency responses and N acquisition, thus constituting a complicated mechanism. However, since Arabidopsis NIGT1 proteins can form heterodimers with certain HHO proteins (Ueda et al. 2020b), the NIGT1 subfamily members potentially regulate N acquisition and utilization in a more complex manner than currently understood.

## PHR1-NIGT1 interplay balancing N and P acquisition activities

**Fig. 3:**
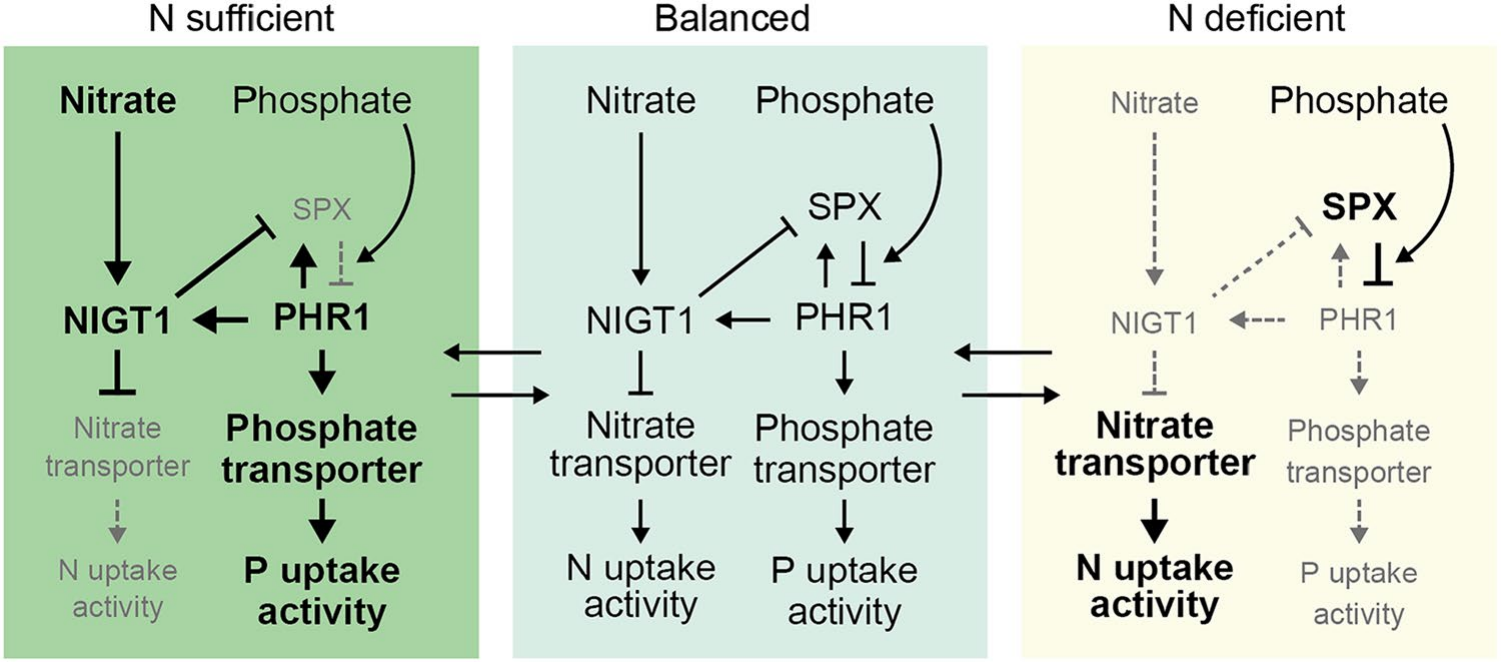
A schematic diagram of the PHR1- and NIGT1-mediated mechanism that balances N and P acquisition. Under N-sufficient conditions, nitrate increases *NIGT1* expression, causing the downregulation of nitrate transporter genes to prevent excess absorption of nitrate. The increase in *NIGT1* expression also represses *SPX* expression, resulting in increased PHR1 activity and upregulation of phosphate transporter genes to coordinate the cellular phosphate level. Then, because of NIGT1 autoregulation and PHR1-dependent activation of *NIGT1* and *SPX1* genes for positive and negative feedback regulations, the expression levels of these genes reach a steady state. Because SPX proteins suppress PHR1 activity in response to the cellular phosphate level, phosphate also participates in this regulatory network. Under N-deficient conditions, *NIGT* expression is decreased, inducing the upregulation of nitrate transporter genes, followed by increased N uptake activity. The decrease in *NIGT* expression also de-represses the *SPX* genes, which results in reduced PHR1 activity and phosphate uptake to prevent the excess uptake of phosphate relative to that of nitrate. The modulation of the NIGT1–SPX–PHR cascade in response to cellular nitrate and phosphate levels causes mutual activation and repression among these factors, optimizing the balance of nitrate and phosphate. The upregulated and downregulated signals and factors are indicated by bold font and thick lines in black and small letters and dashed lines in gray, respectively

N and P are key soil nutrients necessary in exceedingly high amounts, because they constitute various biomolecules. Plants must allocate resources to either the nitrate uptake pathway or the phosphate uptake pathway, depending on the demand and availability of these nutrients, to achieve optimal growth in different nutrient environments without wasting energy. Thus, plants need a complicated system to balance N and P acquisition activities. In recent years, the mechanisms underlying balanced nutrient acquisition under diverse nutrient conditions have emerged as a new research target (Oldroyd and Leyser 2020). Recent studies on Arabidopsis NIGT1 and PHR1 subfamily members revealed a new mechanism, which indicated that both NIGT1 and PHR1 TFs constitute the core part of the transcriptional regulatory network that coordinates N and P acquisition (Maeda et al. 2018; Ueda et al. 2020a). In the proposed model (Fig. 3), nitrate induces *NIGT1* expression to downregulate nitrate transporter genes and subsequently prevent the excess absorption of nitrate under N-sufficient conditions. Simultaneously, NIGT1 binds to the *SPX* gene promoters and represses *SPX* expression to enhance PHR1 activity and phosphate uptake activity, thereby eliminating the problem of lower phosphate uptake activity compared to nitrate uptake activity. The expression or activity of NIGT1, SPX, and PHR1 reaches a steady state when mutual activation and repression among these factors leads to an optimal balance of nitrate and phosphate uptake activity. At this stage, *NIGT1* expression is under the control of autorepression, owing to the presence of NIGT1-binding sites in *NIGT1* gene promoters, probably contributing to maintaining this steady state. By contrast, under N-deficient conditions, plants must balance the amounts of acquired N and P by promoting nitrate uptake and decreasing phosphate uptake. Consistent with this requirement, N deficiency reduces *NIGT1* expression levels, leading to higher nitrate uptake activity. At the same time, N deficiency*-*repressed *NIGT1* expression upregulates *SPX* expression, reducing PHR activity to suppress phosphate uptake activity. This model is consistent with the root development phenotypes of *hrs1* (*nigt1.4*) mutants and *HRS1* (*NIGT1.4*) overexpressors under phosphate deficiency conditions (Liu et al. 2009; Medici et al. 2015; Nagarajan et al. 2016). Conversely, PHR1 and PHL1, activated in response to phosphate starvation, directly enhance *NIGT1* expression to reduce the expression of nitrate transporter genes and consequently decrease nitrate uptake activity to a level proportional to phosphate uptake. Since one of the NIGT1-binding sequences (-GAATATTC-) is a variation of P1BS (-GNATATNC-), NIGT1 and PHR1 proteins co-regulate *NIGT1* expression using shared binding sites (Maeda et al. 2018). However, because of their unshared binding sites, NIGT1 and PHR1 can also regulate distinct target genes individually to precisely balance N and P acquisition.

The transcriptional network described above can explain the mechanism that balances N and P acquisition. However, the precise mechanism may be more complicated than this simplified model, because of the presence of multilayered positive and negative regulations such as the autorepression of NIGT1, activation of *SPX* genes by PHR1, and PHR1–SPX complex formation-mediated repression of PHR1 activity. Medici et al. (2015) also reported that the stability of NIGT1.4/HRS1 is decreased by phosphate deficiency, although how its stability is regulated post-translationally remains to be elucidated. Furthermore, Hu et al. (2019) reported that OsSPX4-mediated regulation of OsPHR2 subcellular localization is also involved in balancing nitrate and phosphate acquisition in rice. Under phosphate-deficient conditions, OsPHR2 is sequestered in the cytosol by OsSPX4 via direct interaction to suppress phosphate uptake. Hu et al. (2019) proposed that nitrate application enhances OsSPX4 degradation through the activation of an E3 ubiquitin ligase, NRT1.1B-INTERACTING PROTEIN 1 (NBIP1), which allows OsPHR2 to translocate to the nucleus to activate phosphate transporter gene expression.

## Concluding remarks and future perspectives

As plant-specific TFs, the GARP family proteins are involved in various plant-specific physiological processes. Recent studies revealed that PHR1 and NIGT1 subfamily members are key regulators of P and N acquisition. Phylogenetic analysis indicates that PHR1 and NIGT1 subfamily members possessing different amino acid sequences for the CCD do not belong to a monophyletic lineage, suggesting that the PHR1 and NIGT1 subfamily emerged evolutionarily independently (Fig. 1). However, many studies revealed that the interplay of PHR1 and NIGT1 subfamily members constitutes a central regulatory network that integrates the supply and demand information of N and P for optimizing nutrient acquisition. Thus, most GARP family proteins that function as dimeric TFs are likely involved in regulating the nutrient response. Furthermore, given the presence of NIGT1 and PHR1 homologs in a liverwort (Fig. 1b), the transcriptional network mediated by NIGT1 and PHR1 family members appears to be evolutionarily conserved across the entire plant kingdom to regulate nutrient responses. Therefore, further analyses of GARP family proteins, especially PHR1 and NIGT1 subfamily members, would reveal the fundamental mechanism that regulates the acquisition of the most critical soil nutrients (N and P) in plants. Improving the utilization efficiencies of soil nutrients in crops is essential for the development of a sustainable agriculture system with reduced fertilizer usage, and the gene regulatory network regulated by NIGT1 and PHR1 subfamily members is a potential target for such improvements, as already demonstrated through the disruption of *OsHHO3* in rice. Thus, PHR1 and NIGT1 subfamily members remain attractive targets for both basic and applied plant research.
